# Insight into the metabolic mechanism of scoparone on biomarkers for inhibiting Yanghuang syndrome

**DOI:** 10.1038/srep37519

**Published:** 2016-11-21

**Authors:** Heng Fang, Aihua Zhang, Jingbo Yu, Liang Wang, Chang Liu, Xiaohang Zhou, Hui Sun, Qi Song, Xijun Wang

**Affiliations:** 1 Sino-America Chinmedomics Technology Cooperation Center, Chinmedomics Research Center of TCM State Administration, National TCM Key Laboratory of Serum Pharmacochemistry, Department of Pharmaceutical Analysis, Laboratory of Metabolomics, Heilongjiang University of Chinese Medicine, Heping Road 24, Harbin 150040, China; 2 State Key Laboratory of Quality Research in Chinese Medicine, Macau University of Science and Technology, Avenida Wai Long, Taipa, Macau

## Abstract

Scoparone (6,7-dimethoxycoumarin) is the representative ingredient of Yinchenhao (*Artemisia capillaris* Thunb.) which is a famous Chinese medicinal herb and shows favorable efficacy for all kinds of liver disease, specifically for the treatment of Yanghuang syndrome (YHS). The precise molecular mechanism concerning the action of scoparone on YHS is yet to be fully elucidated. The aim of the present study was to determine the mechanism of scoparone and evaluate its efficacy on metabolite levels. The differential expression of metabolites responsible for the pharmacological effects of scoparone was characterized and the protection effect of scoparone against this disease. Using multivariate statistical analysis, 33 biomarkers were identified using precise MS/MS and play an important role in the regulation of key metabolic pathways associated with liver disease. In addition, pathological results also showed consistent changes in the YHS model group and after treatment with scoparone, both the metabolic profile and histopathology resembled that of normal level, which suggesting favorable efficacy over the observed time period. The present work indicated that a metabolomics platform provided a new insight into understanding the mechanisms of action of natural medicines such as scoparone.

Natural medicinal products are recognized as significant resources which provide reliable modern medicines. The wide application of Artemisinin^1^, demonstrates a typical example of how a nature medicine can be utilized and has attracted much attention in the medical community. Scoparone (Supplemental Fig. 1) is an important type of coumarins which exist in many natural substances^2^ such as *Artemisia argyi Levl.et* Vant., *Pulsatilla chinensis* (Bge.) Regel, *Lobelia chinensis* Lour., *Puerarua lobata* (Willd.) Ohwi and *Artemisia capillaris* Thunb. Modern pharmacological studies have shown it is a major constituent of *Artemisia capillaris* Thunb. that is quickly absorbed and slowly eliminated^3^,^4^,^5^. For thousands of years, *Artemisia capillaris* Thunb. was used as the dominant herb in the treatment of jaundice in Asia and showed remarkable hepato-protective ability and a choleretic effect^6^,^7^. However, the precise mechanism underpinning the treatment of jaundice has not yet been reported which seriously limits the development of drug discovery in this area.

Metabolomics is a novel field within the omics sciences which has much potential to impact scientific discovery as a powerful technique to investigate biological phenomenon. Currently, traditional clinical biochemical indexes are used for typical diseases or organ injuries which are not suitable for the diagnosis of complex systems, especially for traditional Chinese medicine syndrome (CMS)^8^,^9^,^10^,^11^,^12^. The practicable characterization of CMS needs to be represented by groups of specific biomarkers of patients which possess irreplaceable positions in regulating associated metabolism. Only with an improved overall understanding from multi-level networks or multiple targets can the mechanism of CMS be fully determined.

YHS is an exclusive syndrome in CMS recorded in the classic monograph of traditional Chinese medicine (TCM) named ‘Shang han lun’. YHS patients show bright yellow coloring in the skin and sclera which is also a distinguishing feature between Yanghuang and Yinhuang syndromes. Unlike jaundice syndrome, YHS indicates pathological changes in patients and it has significant potential for improved diagnosis and therapy. According to the theory of TCM pathogenesis, we established an YHS model by orally administered with *Zingiber officinale* Rosc., ethanol, and α-naphthylisothiocyanate (ANIT). An advanced UPLC-Q/TOF-G2Si-HDMS system, robust data processing platform and comprehensive network analysis were used to determine the pathogenesis of YHS and pharmacodynamic evaluation of scoparone.

## Results

### Histopathological results

YHS patients generally experience liver disease^13^,^14^,^15^,^16^,^17^,^18^,^19^,^20^,^21^,^22^ as a direct result of disordered liver metabolism. From the histopathological observations, a significant change in the microscopic histology of H&E stained liver sections was detected in the YHS group. Specifically, the histology showed regional laminar necrosis and edema around the central vein combined with inflammatory cell infiltration in the liver lobules. Compared with YHS group, the scoparone group showed partial remission with the treatment (Supplemental Fig. 2).

### Metabolite identification and metabolic pathway analysis

**Figure 1:**
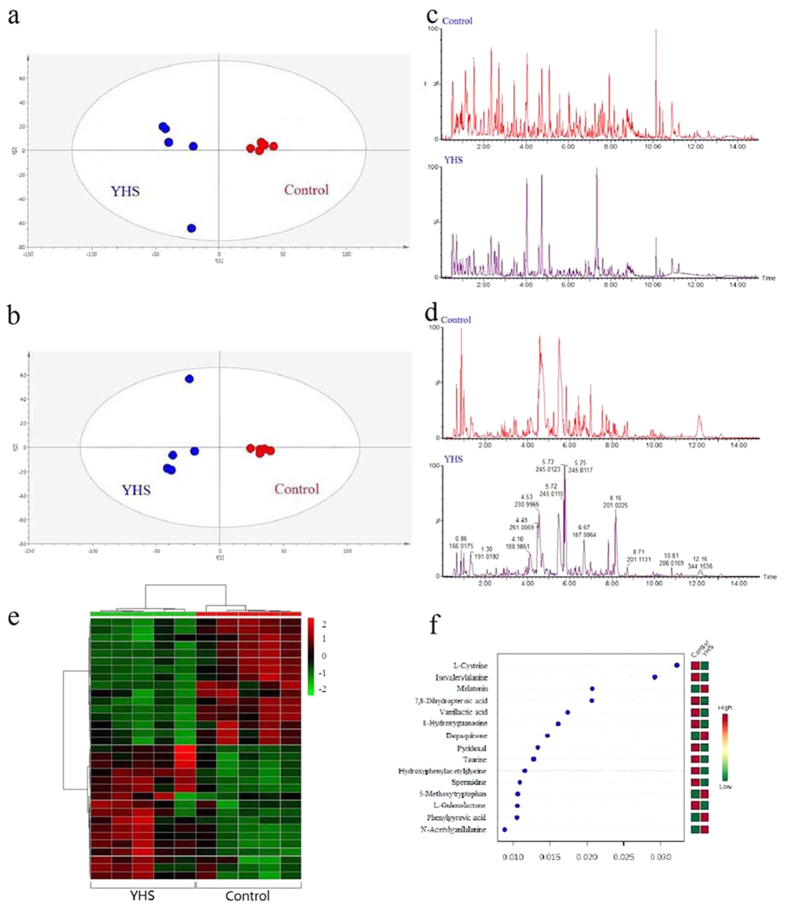
Pattern recognition analysis of the UPLC/MS spectra of urine samples. (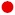) Control group and (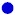) YHS group in positive mode (**a**) and negative mode (**b**). Fingerprint of the control group and YHS group in positive mode (**c**) and negative model (**d**). Heat map visualization for YHS mice in positive mode and negative mode (**e**). The top 15 significant features of the metabolic markers based on the VIP projection (**f**).

**Figure 2:**
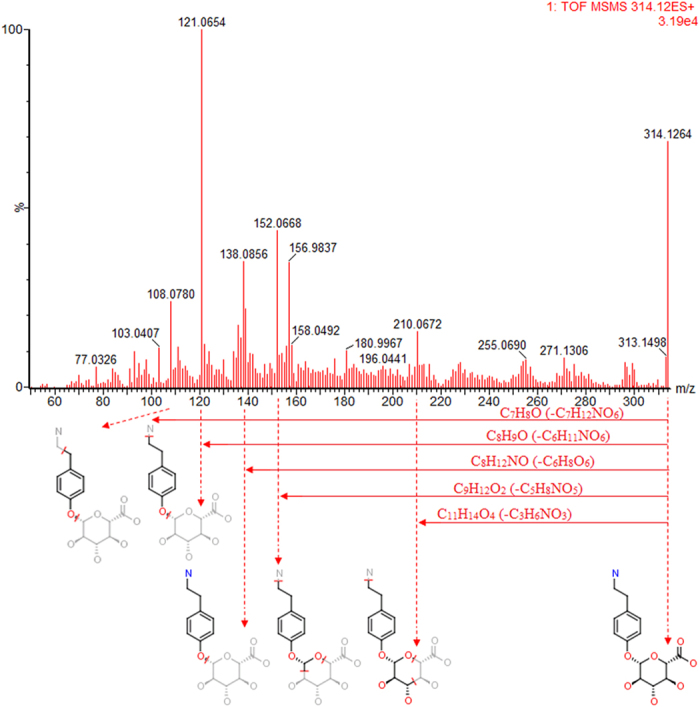
Identification of the chemical structures and mass fragments of tyramine glucuronide.

A total of 33 ions (VIP > 1, *t test* < 0.05) were recognized as potential novel biomarkers of response across the experimental groups (Fig. 1, Table S1). Amongst these, 30/33 biomarkers were decreased to a level similar to that of the control level (Supplemental Fig. 3). With high accurate scanning of MS and MS/MS signals and the statistical screen, the ions (Rt = 1.96 min, m/z determined = 314.1240) were demonstrated as potential molecules for the additional identification. Based on the possible chemical bond breaking and the comprehensive information of HMDB, ChemSpider and KEGG, final identification was accurately matched with tyramine glucuronide (Fig. 2). These low molecular weight metabolites were involved in multiple pathways and reactions including pentose and glucuronate interconversions, taurine and hypotaurine metabolism, primary bile acid biosynthesis and glutathione metabolism. Some of the metabolites showed a high correlation with bilirubin metabolism suggesting a strong mechanistic role in the development of jaundice.

### Effects of Scoparone against YHS

**Figure 3:**
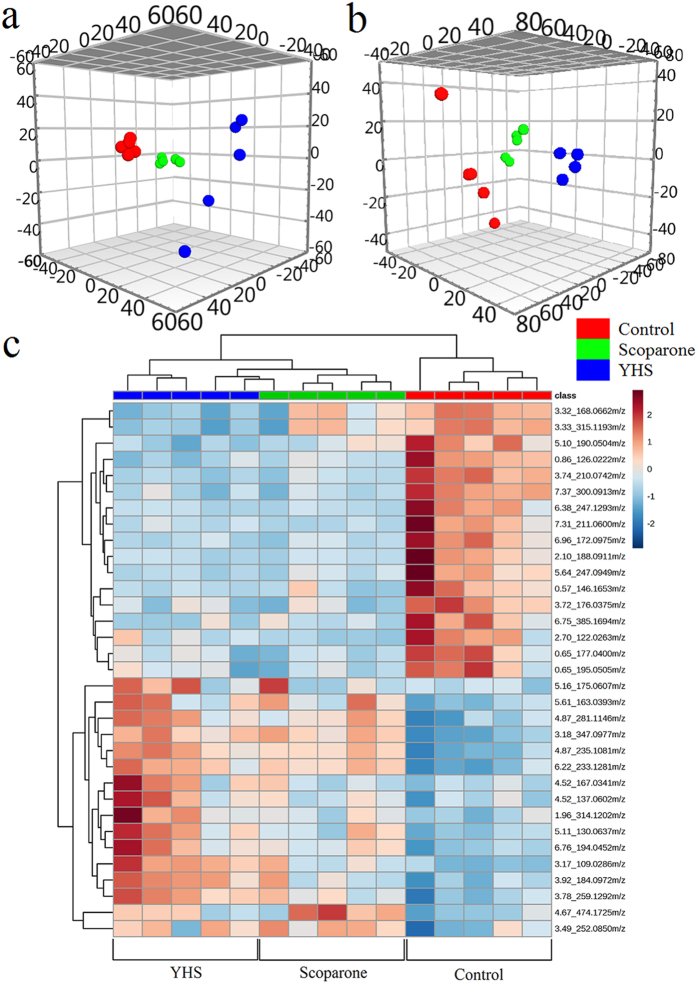
Multivariate analysis of UPLC/MS datasets acquired for different group samples. Score plots for the (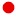) Control group, (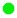) YHS group and (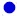) Scoparone groups in positive mode (**a**) and negative mode (**b**). Heat map visualization for the treatment of scoparone on YHS in positive mode and negative mode (**c**).

**Figure 4:**
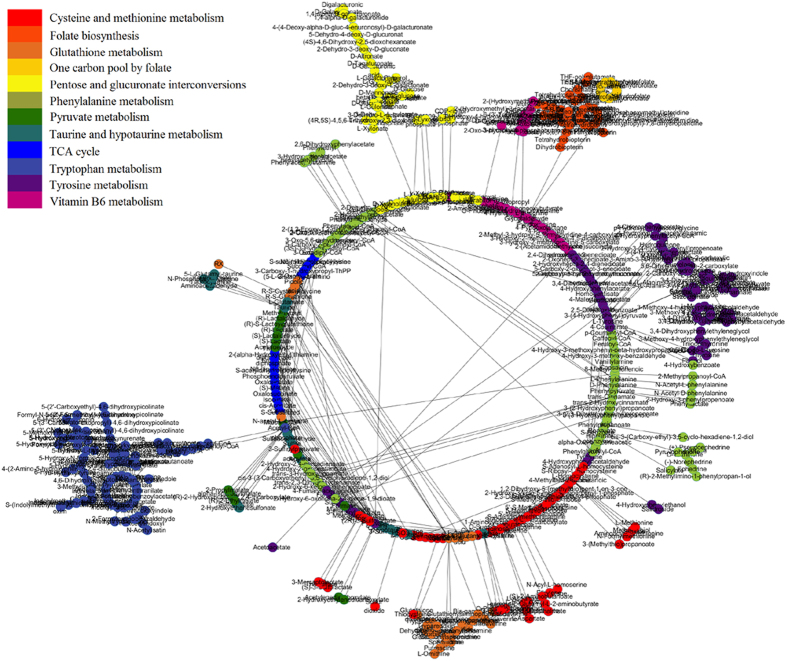
An overview of the perturbed metabolic networks in response to YHS according to KEGG analysis.

**Figure 5:**
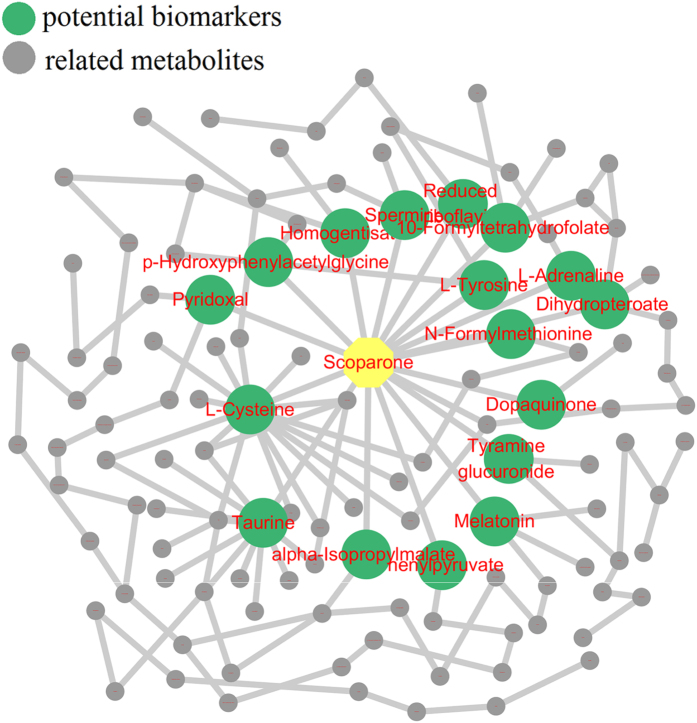
Scoparone regulates marker metabolites of putative effects based on KEGG analysis.

From the metabolic profiling analysis in the three groups (control, YHS and scoparone groups), scoparone group showed an inverse trend in both positive and negative modes (Fig. 3). The above description showed that scoparone exerted a significant effect on YHS mice where the global state appeared to return to the normal level. Potential biomarkers can represent the metabolism of YHS accurately. With the exploration of relevant metabolic pathways, a metabolic network for the potential biomarkers and pathways based on comprehensive network analysis was built (Fig. 4). Amongst these, potential enzymes and reactions were discovered which included pentose and glucuronate interconversions, taurine and hypotaurine metabolism, primary bile acid biosynthesis and glutathione metabolism. The involvement of these pathways suggests a potential mechanism for the effects of YHS. It is well known that increased levels of bilirubin directly lead to the skin and sclera xanthochromia, ultimately leading to the occurrence of jaundice. During the metabolism of bilirubin, UDP-glucuronosyltransferase 1A1 plays an important role in transformation from hydrophobicity to hydrophilicity of pentose and glucuronate interconversions. This metabolic process has major biological significance for detoxification and excretion of metabolites including tyramine glucuronide (Fig. 5). After treatment with scoparone, these biomarkers all showed a significant decrease.

### Correlation analysis of biomarkers between clinical samples and the YHS mice model

CMS cannot be diagnosed using the traditional biochemical index. The emerging metabolomics methods used in this study possessed a comprehensive and dynamic viewpoint which is a more suitable approach for the characterization of CMS. Focusing on biomarkers which have potential roles in the regulation of metabolism may reveal the underlying biological mechanism. In a previous study from our group^23^, we have reported urine metabolomic analysis for biomarker discovery and detection of jaundice syndromes in patients with liver disease, the results indicated that metabolites contributed to the complete separation of jaundice from matched healthy control patients. These biomarkers were used for analysis of the YHS mice model. Using comparative analysis of biomarkers between clinical samples and the YHS mice model, ten common biomarkers were identified as the same molecular structure which had interaction in key pathway associated with YHS, strongly suggesting the established YHS mice model is highly specific and relevant for the study of the human condition.

## Discussion

YHS is considered as a complex CMS which cannot be accurately diagnosed using clinical chemistry and pathology. These evaluation indicators were established on the basis of disease state and organ injury, which do not comprehensively reflect the characteristic features of CMS. The TCM theory for the classification of jaundice diagnosis is crucial to the diagnosis and treatment of the specific disease or CMS and is a predecessor for precision medicine approaches in modern medicine and suggests the rationality of YHS. Considering a lack of robust advanced methods, the recognition of CMS has been limited. Giving the emerging strengths of metabolomics, the technology has much potential as robust tool for the classification of CMS.

The present study constructed the YHS mice model considering this advanced discipline and previous investigations. From the histopathology, regional laminar necrosis and edema were found to exist around the central vein with inflammatory cells infiltration indicating serious injury in the liver of YHS mice. Phenotypic characterization of YHS was performed using UPLC-G2Si-HDMS technology combined with pattern recognition methods. Having determined the optimum data mining approach with ultra-modern software and precise MS/MS fragments of non-targeted ions, ten key metabolites were identified to be identical to the structural and biological markers of jaundice patients. These potential biomarkers play a pivotal role in regulation of associated metabolic networks.

**Figure 6:**
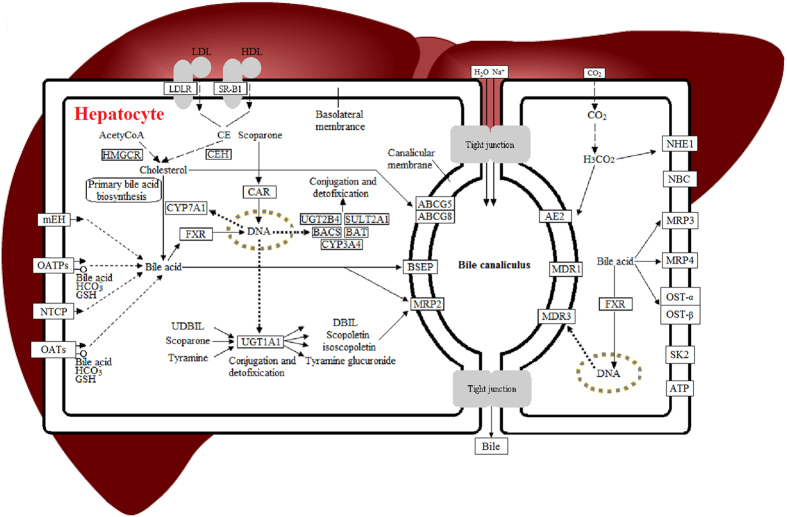
The proposed mechanism of scoparone protection against YHS.

Tyramine glucuronide, a derivative of tyramine, is a major terminal end product which can induce some kind of anaphylaxis including urticaria and asthma. It originates from a wide range of food sources and needs to be excreted or metabolized daily by hepatocytes. From pentose and glucuronate interconversions, tyramine glucuronide is produced from the transformation of tyramine glucuronidation by UDP-glucuronosyltransferase 1A1 [EC2.4.1.17]. It is well known that the liver possesses irreplaceable metabolic functions including detoxification capacity. Amongst thousands of metabolic enzymes, UDP-glucuronosyltransferase 1A1 is the dominant enzyme involved in the clearance of xeno-chemicals, drugs and endogenous substances^24^,^25^ which influence the metabolism and transport of bilirubin. With the specific biotransformation of UDP-glucuronosyltransferase 1A1, the hydrophilic property of bilirubin occurs as a structural transformation, which is conducive to excretion with urine and feces. As YHS is fundamentally a liver system disease which is often accompanied with metabolic disturbance of enzymes, unconjugated bilirubin reflows into blood with the combination of elasticin ultimately results in skin and sclera xanthochromia. For a short time, bilirubin accumulation causes the development of a bright color which is diagnosed as YHS. The present work found the content of tyramine glucuronide was reduced in the YHS group suggesting disordered bilirubin metabolism of bilirubin occurring simultaneously. After treatment with scoparone it was gradually observed to recover to the normal level. Several investigations have shown that scoparone is the ligand of the constitutive androstane receptor (CAR)^26^,^27^,^28^,^29^,^30^ (Fig. 6). As for a UDP-glucuronosyltransferase 1A1 agonist such as phenobarbital, scoparone may improve the action of CAR as a transcription factor to control the expression of the UDP-glucuronosyltransferase 1A1 gene. Furthermore, tyramine glucuronide as a potential biomarker may be more sensitive and accurate for clinical diagnosis of YHS. While, from the targeted verification of ELISA kit for UDP glucuronosyltransferase 1A1, an obviously callback trend was represent in the detection of hepatic tissue (Supplemental Figure 4).

Taurine is the precursor compound of taurocholate, which is an important constituent of bile in primary bile acid biosynthesis. It is known that taurocholate is a necessary element for the absorption of lipids in the digestive tract which relieves the obstruction of bile by increasing the solubility of lipids and cholesterol. The clinical manifestation of YHS is strongly associated with bile metabolism. In taurine and hypotaurine metabolism, taurine is derived from L-cysteine under the regulation of glutamate decarboxylase [EC4.1.1.15]. Modern pharmacological studies have shown that the activity of glutamate decarboxylase directly influences the metabolism of taurine and may cause the occurrence of cerebral palsy, which is associated with hyperbilirubinemia^30^,^31^,^32^. The taurine pathway is therefore a potential factor involved in YHS pathogenesis. Following treatment with scoparone, taurine levels recovered to be normal levels which further supports the finding that secretion of bile and bilirubin metabolism were partially relieved.

Phenylpyruvic acid is a direct metabolite of phenylalanine which also an essential amino acid. Tyrosine aminotransferase [EC2.6.1.5] is the enzyme which is necessary for the conversion of phenylalanine to phenylpyruvic acid. Disorders of enzyme activity also induce tyrosinemia which is clinically associated with jaundice. Partial injury to liver cells injury may cause aspartate transaminase [EC2.6.1.1] to be released into blood which is eventually detected as an important index indicating liver function. In this study, we found the level of phenylpyruvic acid in YHS was significantly higher than that of the control group, suggesting that key metabolites and enzymes were significantly altered and resulted in a high level of morbidity associated with jaundice. Following treatment with scoparone, the content of phenylpyruvic acid decreased to a level similar to that of the control group indicating scoparone had a specific function on the regulation of phenylpyruvic acid.

Glutathione metabolism is the most common reaction in many cellular reactions. With the regulation of vital metabolites or enzymes, natural biological barriers could play a fundamental protection role including broad-spectrum detoxification, anti-inflammatory response, antioxidation and antitumor activity. Remarkably, it is also used for the diagnosis of liver disease. There were a series of enzymes that dominate vital biological actions such as glutathione disulfide (GSSG), glutathione (GSH), glucose-6-phosphate dehydrogenase [EC1.1.1.49] and glutathione peroxidase [EC1.11.1.9]. In this study, we found the content of spermidine and L-cysteine was significantly reduced in the YHS group which suggests the activity of GSH and GSH-Px [EC1.11.1.9] was inhibited below the normal level. Glucose-6-phosphate dehydrogenase deficiency may also cause neonatal jaundice^33^. Through the combination of clinical chemistry information and metabolomics data, it can be concluded that the glutathione metabolism is altered beyond the normal level and this may ultimately developed into YHS.

## Conclusion

In this study, a powerful metabolomics platform was constructed to interpret the pathogenesis of YHS and the mechanism of action of scoparone. Using non-invasive collection methods, biological data were obtained under optimum conditions. In addition, we employed previously reported biomarkers to perform the urine metabolomic analysis of jaundice syndrome in patients as an important standard to investigate YHS mice. Pathway analysis revealed a series of key enzymes and metabolites that were directly associated with the pathogenesis of YHS. From these associated changes, we focused on core pathways including pentose and glucuronate interconversions, taurine and hypotaurine metabolism, phenylalanine, tyrosine and tryptophan biosynthesis. The discovered biomarkers had potential value for the diagnosis of YHS, some of which were also the key targets for scoparone treatment.

In summary, based on pathogenesis and TCM theory, for the first time we established a novel YHS mice model and used detailed pathway analysis to compare clinical YHS biomarkers and verify the rationality of the model. Our work suggests that urine metabolomics as a robust platform to explore the pathogenesis of CMS has much potential in elucidating response mechanisms of scoparone.

## Materials and Methods

### Chemicals and reagents

Acetonitrile of high performance liquid chromatography was purchased from Merck (Darmstadt, Germany). Ultrapure water was further purified using a Milli-Q system (Millipore, Bedford, MA, USA). α-naphthylisothiocyanate (ANIT) was purchased from Sigma-Aldrich (MO, USA). *Zingiber officinale* Rosc. was purchased from Harbin Tong Ren Tang Drug Store (Harbin, China). Alcohol was purchased from Beijing Reagent Company (Beijing, China). Scoparone (purity 99%) was purchased from Sichuan Provincial Institute for Food and Drug Control (Sichuan, P. R. China). ELISA kit for UDP-glucuronosyltransferase 1A1 (Shanghai Shuangying Biological Technology Co.,Ltd, China).

*Zingiber officinale* Rosc. (50.8 g) was decocted in 500 mL distilled water for 1 h and then filtered through 6 layers of gauze. The residue was added into 10 times the volume of distilled water for being decocted for 1 h. The process was repeated 3 times before combining three filtrates for furthering concentration to 500 mL. 15.6 mL was added to 104.4 mL of distilled water to prepare the orally administered solution at the concentration of 0.013 g/mL.

### Sample collection and preparation

Male Balb/c mice (weighting 20 ± 2 g) were supplied by the GLP center of Heilongjiang University of Chinese Medicine (Harbin, China). Mice had free access to food and water with comfortable housing conditions at a temperature of 24 ± 1 degree Celsius and 60 ± 5% humidity. Prior to the urine collection, mice were allowed to acclimatize to metabolic. They were then randomly divided into three groups with five mice in each group. These were control, YHS and scoparone groups. The YHS and scoparone groups were given orally administration of *Zingiber officinale* Rosc. extracted solution (0.013 g/mL) in alcohol (3.125% (v/v) at 8 a.m and 2 p.m at a dose of 0.1 mL/10 g for fourteen days. Mice from the control group were orally administered distilled water at the same dose per day. From days fifteen and sixteenth, the YHS group was given different concentrations of ANIT solution (1.5 mg/mL, 1 mg/mL, dissolved in olive oil) at 2 p.m at a dose of 0.1 mL/10 g once per day. On the seventeenth day, the scoparone group was treated with 50 mg/kg for seven days. The control group received orally administered olive oil at the same dose once day.

Urine samples were collected from metabolism cages at 8 a.m and immediately centrifuged (4 degree Celsius, 15 min, 13000 rpm) to remove impurities. Supernatants were stored at −80 degree Celsius until metabolomic analysis was performed. At the end of the experiment, all mice were sacrificed and liver tissues were collected and fixed in 10% formalin for histopathology analysis. The above investigation was approved by the Ethical Committee of Heilongjiang University of Chinese Medicine and was conducted according to the principles expressed in the Declaration of Helsinki.

### Histopathology analysis

To observe the histopathological changes in YHS mice, fresh liver tissues were immediately delivered to the affiliated hospital of Heilongjiang University of Chinese Medicine for conducting the histopathology analysis.

## UPLC/MS experiments

### Chromatography

We employed an ultra-high performance liquid chromatography (UPLC) system (Waters Corp., Milford, MA/USA) for the global analysis of urine samples using MassLynx^TM^ software (V4.1 SCN901). A brand-new Acquity UPLC HSS T3 Column (100 mm × 2.1, 1.8 μm; Waters Corporation, Milford, USA) was used for separation. After the comprehensive exploration of chromatography conditions, an optimum method was used for the data collection under linear gradient conditions consisting of: (A) acetonitrile with 0.1% formic acid and (B) water with 0.1% formic acid as the mobile phase, and a flow rate set at 0.4 mL/min. The gradient eluting condition was: 1 to 10% A, 0–3 min; 10 to 20% A, 3–5 min; 20–40% A, 5–8.5 min; 40 to 99% A, 8.5–9.5 min; maintaining 99% A at 9.5–11.5 min; 11.5–12 min, linearly decreasing from 99% to 1% A; held at 1% A for 3 min for equilibration of the column. The sample injection volume was 2 μL. To optimize and ensure the stability and reproducibility of the UPLC-Q/TOF-G2Si-HDMS system, we employed a quality control (QC) specimen from each group which contained the most information of all urine samples during the whole process (Supplemental Figure 5).

### Mass spectrometry

The high throughput G2Si High-definition mass spectrometry (Waters Q-TOF SYNAPT^™^, Waters Corp, Manchester, England) with electrospray ion source was used for global detection of biological information. The optimum parameters were as follows: both positive and negative modes were operated at a capillary voltage of 3 kV and a cone voltage at 30 V. The desolation gas flow rate was maintained 800 L/h with a cone gas flow of 50 L/h. The desolation temperature was 350 degree Celsius and a source temperature at 110 degree Celsius was used. Date detected in centroid mode from 50~1200 Da. To ensure precise and stable scanning, a lock-mass of leucine enkephalin at a concentration of 0.2 ng/mL was used as a lock spray interface at a flow rate of 100 mL/min ([M + H]^+^ =556.2771, [M-H]^−^ = 554.2615).

### Data preprocessing and Multivariate data analyses

All raw data were imported into the Progenesis QI software (Nonlinear Dynamics, 2014, version 1.0) for preprocessing. The robust and intelligent system is capable of processing hundreds of data files at one time. Before multivariate analyses, data preprocessing was the core procedure which contained noise reduction, normalization and peak picking. The present investigation employed 50000 as the full width at half maximum according to the performance of G2Si-HDMS, then series of possible adducts selected in corresponding ionisation polarity. Besides, the automatic sensitivity method was used as a noise estimation algorithm to determine the noise levels in the data. The higher the sensitivity value, the more compound ions will be detected. Chromatographic peak width was not applied for a minimum peak width. With a narrow mass error of 5 ppm, the interesting ions would be identified accurately. A multidimensional matrix from EZinfo 2.0 software was generated for multivariate analysis which included principal component analysis (PCA) and orthogonal partial least squares discriminant analysis (OPLS-DA). From these analyses, the variable importance in projection was selected as the standard for screening.

### Identification of biomarkers

Quantification of compounds was conducted with commercial databases using precise MS/MS fragments. Objectively and strictly matching analysis was used with the automatic identification system of the Progenesis QI software based on the above protocols.

### Statistical analysis

SPSS software (Version 18.0 for windows, IBM, Chicago, IL) was used for the statistical analysis of the ions between the control and YHS group. The content of the ions between the two groups was compared to filter the difference, and the *P* value of a Student *t test* was less than 0.05. Combined VIP lists of OPLS-DA and the *p* value of *t test*, series of biomarkers were shown to be differences in metabolites.

## Additional Information

**How to cite this article**: Fang, H. *et al.* Insight into the metabolic mechanism of scoparone on biomarkers for inhibiting Yanghuang syndrome. *Sci. Rep.* **6**, 37519; doi: 10.1038/srep37519 (2016).

**Publisher’s note:** Springer Nature remains neutral with regard to jurisdictional claims in published maps and institutional affiliations.

## Supplementary Material

Supplementary Information
