# Supplementary material for: Insight into the metabolic mechanism of scoparone on biomarkers for inhibiting Yanghuang syndrome
Source: Sci Rep. 2016 Nov 21;6:37519. doi: 10.1038/srep37519 (PMC5116618; doi:10.1038/srep37519)
Supplement: Supplementary Information [file srep37519-s1.doc]

**Insight into the metabolic mechanism of scoparone on biomarkers for inhibiting Yanghuang syndrome**

Heng Fang^1‡^, Aihua Zhang^1‡^, Jingbo Yu^1^, Liang Wang^1^, Chang Liu^1^, Xiaohang Zhou^1^, Hui Sun^1^, Qi Song^1^, Xijun Wang^1,2^*

1. Sino-America Chinmedomics Technology Cooperation Center, Chinmedomics Research Center of TCM State Administration, National TCM Key Laboratory of Serum Pharmacochemistry, Department of Pharmaceutical Analysis, Laboratory of Metabolomics, Heilongjiang University of Chinese Medicine, Heping Road 24, Harbin 150040, China.

2. State Key Laboratory of Quality Research in Chinese Medicine, Macau University of Science and Technology, Avenida Wai Long, Taipa, Macau

*Address correspondence to:

Prof. Xijun Wang

1. Sino-America Chinmedomics Technology Cooperation Center, Chinmedomics Research Center of TCM State Administration, National TCM Key Laboratory of Serum Pharmacochemistry, Department of Pharmaceutical Analysis, Laboratory of Metabolomics, Heilongjiang University of Chinese Medicine, Heping Road 24, Harbin 150040, China.

2. State Key Laboratory of Quality Research in Chinese Medicine, Macau University of Science and Technology, Avenida Wai Long, Taipa, Macau

Tel. & Fax +86-451-82110818

Email: xijunwangls@126.com

^‡^These authors contributed equally to this work.

**Supplementary Table 1.** Identification and trends of change for differential metabolites

| **NO** | **Rt(min)** | **m/z**  **determined** | **m/z**  **calculated** | **Error**  **（mDa）** | **Ion**  **form** | **Molecular**  **Formula** | **Metabolite**  **Name** | **Trend** | **VIP** | **T'TEST** |
| --- | --- | --- | --- | --- | --- | --- | --- | --- | --- | --- |
| 1 | 0.57 | 146.1653 | 146.1657 | -0.4 | [M+H]^+^ | C_7_H_19_N_3_ | Spermidine | ↓ | 1.00 | 0.0069 |
| 2 | 0.86 | 126.0222 | 126.0225 | -0.3 | [M+H]^+^ | C_2_H_7_NO_3_S | Taurine | ↓ | 3.00 | 0.0062 |
| 3 | 1.96 | 314.1202 | 314.1240 | -3.8 | [M+H]^+^ | C_14_H_19_NO_7_ | Tyramine glucuronide | ↓ | 1.13 | 0.0297 |
| 4 | 2.10 | 188.0911 | 188.0923 | -1.2 | [M+H]^+^ | C_8_H_13_NO_4_ | 2-Keto-6-acetamidocaproate | ↓ | 1.39 | 0.0096 |
| 5 | 2.70 | 122.0263 | 122.0276 | -1.3 | [M+H]^+^ | C_3_H_7_NO_2_S | L-Cysteine | ↓ | 1.42 | 0.0001 |
| 6 | 3.17 | 109.0286 | 109.0290 | -0.4 | [M+H]^+^ | C_6_H_4_O_2_ | 1,2-Benzoquinone | ↑ | 1.45 | 0.0143 |
| 7 | 3.32 | 168.0662 | 168.0661 | 0.1 | [M+H]^+^ | C_8_H_9_NO_3_ | Pyridoxal | ↓ | 1.42 | 0.0010 |
| 8 | 3.33 | 315.1193 | 315.1206 | -1.3 | [M+H]^+^ | C_14_H_14_N_6_O_3_ | 7,8-Dihydropteroic acid | ↓ | 1.61 | 0.0006 |
| 9 | 3.74 | 210.0742 | 210.0766 | -2.4 | [M+H]^+^ | C_10_H_11_NO_4_ | Hydroxyphenylacetylglycine | ↓ | 4.90 | 0.0041 |
| 10 | 3.92 | 184.0972 | 184.0974 | -0.2 | [M+H]^+^ | C_9_H_13_NO_3_ | Epinephrine | ↑ | 2.46 | 0.0442 |
| 11 | 4.67 | 474.1725 | 474.1737 | -1.2 | [M+H]^+^ | C_20_H_23_N_7_O_7_ | 10-Formyltetrahydrofolate | ↑ | 1.54 | 0.0060 |
| 12 | 4.87 | 235.1081 | 235.1083 | -0.2 | [M+H]^+^ | C_12_H_14_N_2_O_3_ | 5-Methoxytryptophan | ↑ | 1.38 | 0.0038 |
| 13 | 4.87 | 281.1146 | 281.1137 | 0.9 | [M+H]^+^ | C_13_H_16_N_2_O_5_ | L-beta-aspartyl-L-phenylalanine | ↑ | 1.31 | 0.0284 |
| 14 | 5.10 | 190.0504 | 190.0504 | 0 | [M+H]^+^ | C_10_H_7_NO_3_ | Kynurenic acid | ↓ | 8.04 | 0.0010 |
| 15 | 5.11 | 130.0637 | 130.0657 | -2.0 | [M+H]^+^ | C_9_H_7_N | 3-Methylene-indolenine | ↑ | 1.39 | 0.0011 |
| 16 | 5.64 | 247.0949 | 247.0930 | 1.9 | [M+H]^+^ | C_9_H_14_N_2_O_6_ | 5,6-Dihydrouridine | ↓ | 1.20 | 0.0040 |
| 17 | 6.22 | 233.1281 | 233.1290 | -0.9 | [M+H]^+^ | C_13_H_16_N_2_O_2_ | Melatonin | ↑ | 1.24 | 0.0013 |
| 18 | 6.38 | 247.1293 | 247.1294 | -0.1 | [M+H]^+^ | C_10_H_18_N_2_O_5_ | L-beta-aspartyl-L-leucine | ↑ | 1.02 | 0.0075 |
| 19 | 6.75 | 385.1694 | 385.1685 | 0.9 | [M+H]^+^ | C_19_H_28_O_6_S | 3b,16a-Dihydroxyandrostenone sulfate | ↓ | 1.16 | 0.0364 |
| 20 | 7.37 | 300.0913 | 300.0944 | -3.1 | [M+H]^+^ | C_10_H_13_N_5_O_6_ | 8-Hydroxyguanosine | ↓ | 1.28 | 0.0024 |
| 21 | 0.65 | 177.0400 | 177.0399 | 0.1 | [M-H]^-^ | C_6_H_10_O_6_ | L-Gulonolactone | ↓ | 1.79 | 0.0241 |
| 22 | 0.65 | 195.0505 | 195.0505 | 0 | [M-H]^-^ | C_6_H_12_O_7_ | Gluconic acid | ↓ | 7.42 | 0.0215 |
| 23 | 3.18 | 347.0977 | 347.0992 | -1.5 | [M-H]^-^ | C_15_H_16_N_4_O_6_ | Riboflavin reduced | ↑ | 3.04 | 0.0077 |
| 24 | 3.49 | 252.0850 | 252.0872 | -2.2 | [M-H]^-^ | C_12_H_15_NO_5_ | N-Acetylvanilalanine | ↑ | 1.02 | 0.0003 |
| 25 | 3.72 | 176.0375 | 176.0381 | -0.6 | [M-H]^-^ | C_6_H_11_NO_3_S | N-Formyl-L-methionine | ↓ | 1.23 | 0.0394 |
| 26 | 3.78 | 259.1292 | 259.1292 | -0.2 | [M-H]^-^ | C_11_H_20_N_2_O_5_ | L-gamma-glutamyl-L-leucine | ↑ | 1.31 | 0.0083 |
| 27 | 4.52 | 137.0602 | 137.0603 | -0.1 | [M-H]^-^ | C_8_H_10_O_2_ | Tyrosol | ↑ | 2.08 | 0.0263 |
| 28 | 4.52 | 167.0341 | 167.0344 | -0.3 | [M-H]^-^ | C_8_H_8_O_4_ | Homogentisic acid | ↑ | 3.44 | 0.0036 |
| 29 | 5.16 | 175.0607 | 175.0607 | 0.1 | [M-H]^-^ | C_7_H_12_O_5_ | 2-Isopropylmalic acid | ↑ | 2.37 | 0.0259 |
| 30 | 5.61 | 163.0393 | 163.0395 | 0.1 | [M-H]^-^ | C_9_H_8_O_3_ | Phenylpyruvic acid | ↑ | 2.45 | 0.0040 |
| 31 | 6.76 | 194.0452 | 194.0453 | -0.1 | [M-H]^-^ | C_9_H_9_NO_4_ | Dopaquinone | ↑ | 1.87 | 0.0024 |
| 32 | 6.96 | 172.0975 | 172.0974 | 1.7 | [M-H]^-^ | C_8_H_15_NO_3_ | Isovalerylalanine | ↑ | 11.8 | 0.0015 |
| 33 | 7.31 | 211.0600 | 211.0606 | -0.6 | [M-H]^-^ | C_10_H_12_O_5_ | Vanillactic acid | ↓ | 1.03 | 0.0104 |

Note: ↑, ↓ compared with the control group; Among them, the molecular structure of biomarkers of number 10, 12, 13, 14, 16, 19, 22, 25, 26, 30 are the same with clinical biomarkers.


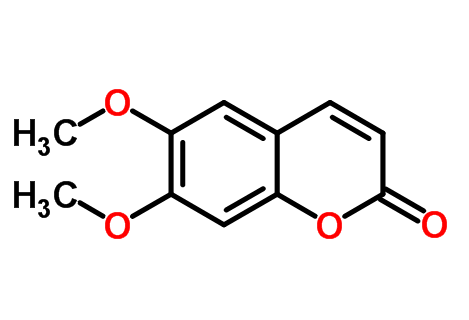


**Supplemental figure 1**. The chemical structure of scoparone.


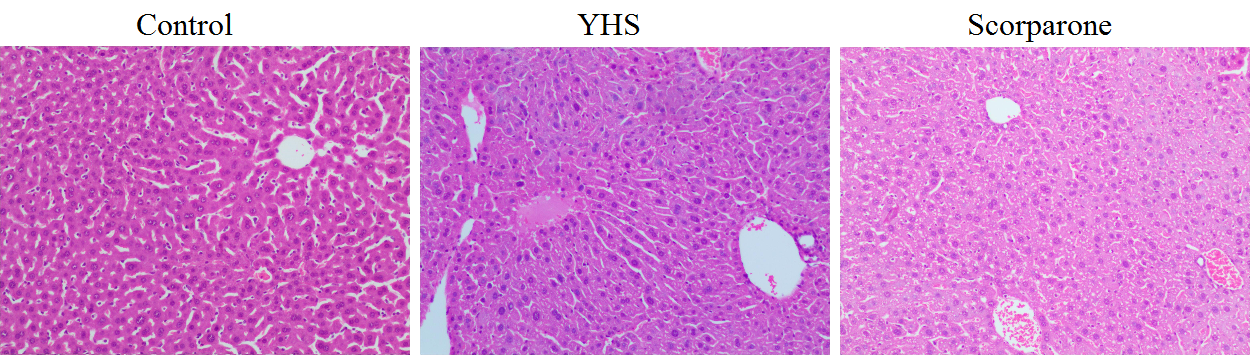


**Supplemental figure 2**. H&E staining for histological evaluation. Typical photographs of hepatic sections stained with H&E. (Magnification 100×).


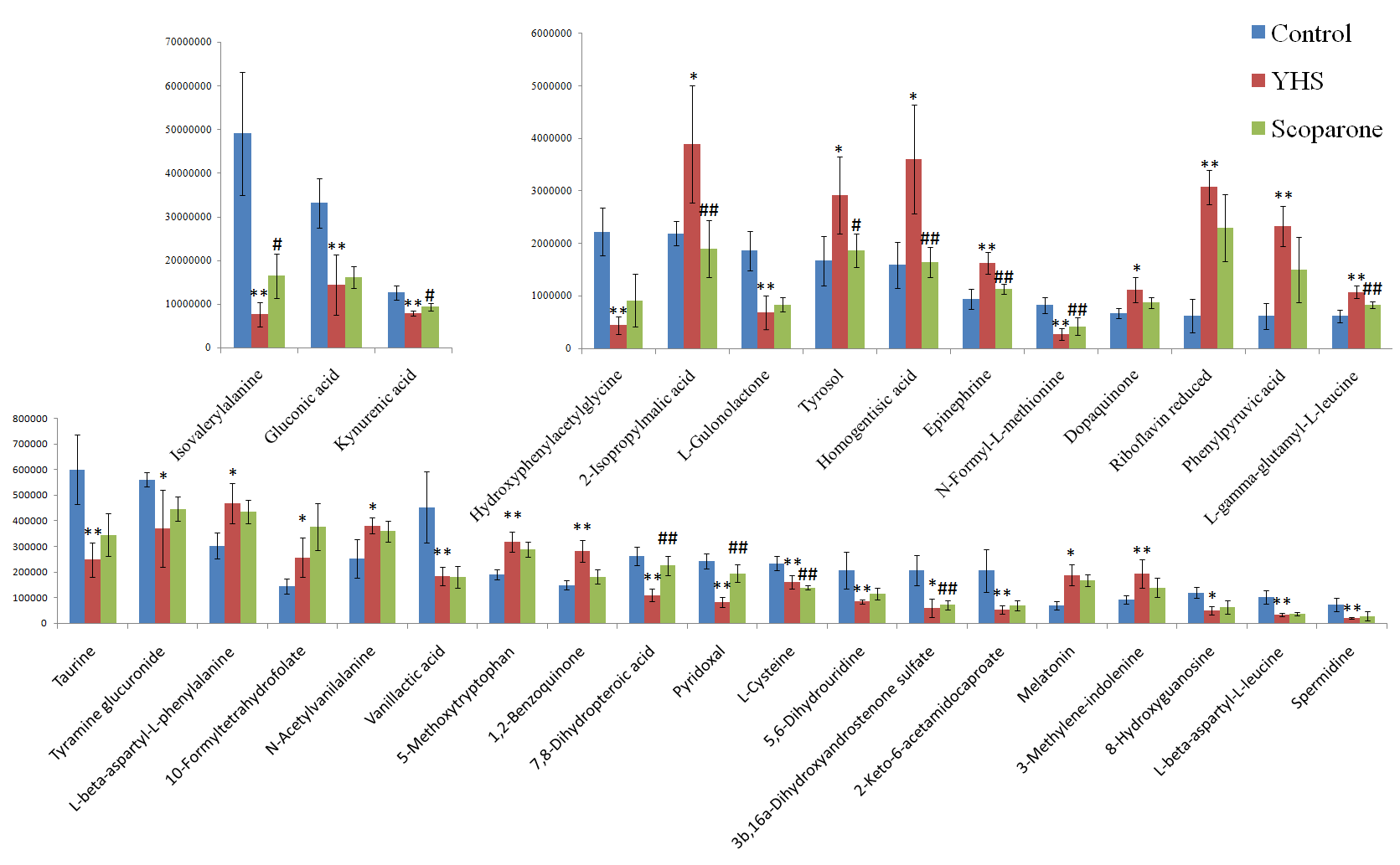


**Supplemental figure 3.** Relative signal intensities of metabolic biomarkers identified by UPLC/MS. Bar plots represent the relative intensities of the 33 metabolites. Data are expressed as mean ± SD. YHS group compared with control group: *p<0.05, **p<0.01 and scoparone group compared with model group: #p<0.05, ##p<0.01.


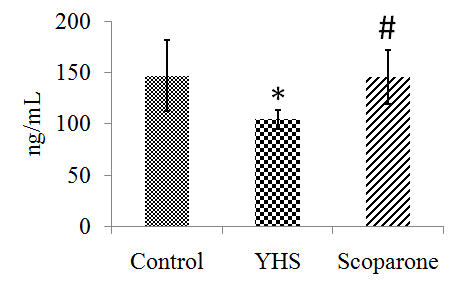


**Supplemental figure 4. The targeted verification of ELISA kit for UDP glucuronosyltransferase 1A1.** Data are expressed as mean ± SD. YHS group compared with control group: *p<0.05, **p<0.01 and scoparone group compared with model group: #p<0.05, ##p<0.01.


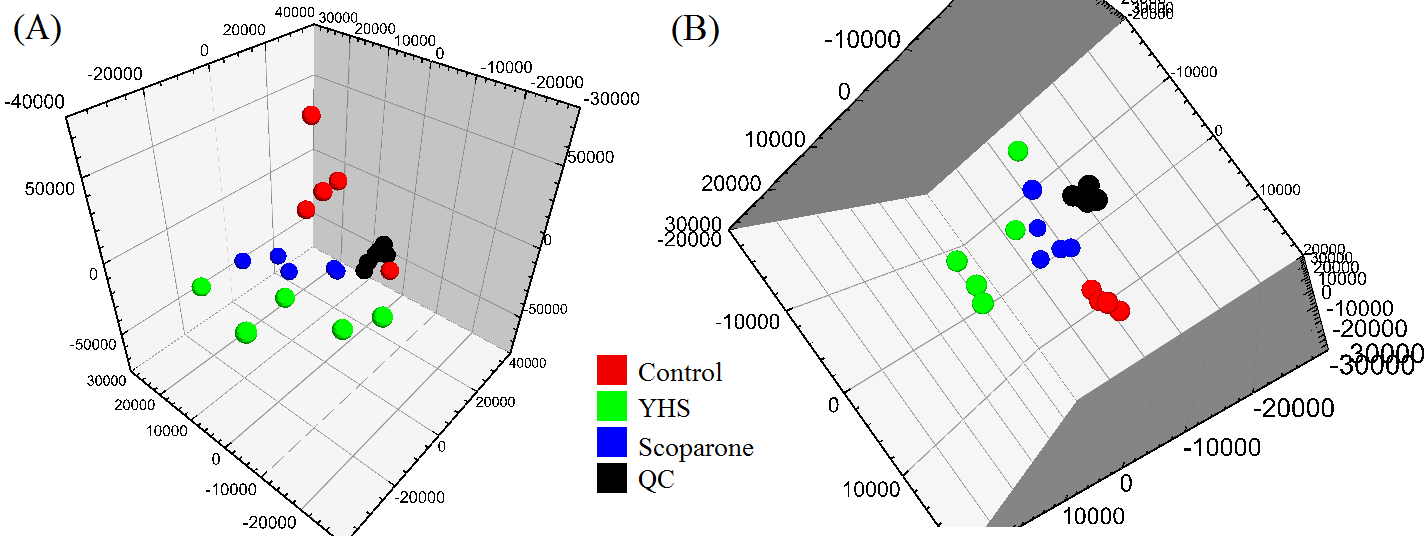


**Supplemental figure 5.** Multivariate data analyses of the UPLC-Q/TOF-G2Si-HDMS urine spectra data with QC samples of 3 D plot of (●) Control group, (●) YHS group, (●) Scoparone group and (●) QC samples in positive mode(A) and negative mode (B). From the analysis of PCA score plot, a tightly cluster was present in the whole dataset, which suggest the instrument and established method possessed a favorable state.
